# Folate and Vitamin B_12_-Related Biomarkers in Relation to Brain Volumes

**DOI:** 10.3390/nu9010008

**Published:** 2016-12-24

**Authors:** Nikita L. van der Zwaluw, Elske M. Brouwer-Brolsma, Ondine van de Rest, Janneke P. van Wijngaarden, Paulette H. In ’t Veld, Daniella I. Kourie, Karin M. A. Swart, Anke W. Enneman, Suzanne C. van Dijk, Nathalie van der Velde, Roy P. C. Kessels, Paul A. M. Smeets, Frans J. Kok, Rosalie A. M. Dhonukshe-Rutten, Lisette C. P. G. M. de Groot

**Affiliations:** 1 Division of Human Nutrition, Wageningen University, Box 8129, 6700 EV Wageningen, The Netherlands; nikitavanderzwaluw@gmail.com (N.L.v.d.Z.); ondine.vanderest@wur.nl (O.v.d.R.); PHjanneke.vanwijngaarden@gmail.com (J.P.v.W.); paulette.intveld@gmail.com (P.H.I.V.); daniella_kourie@hotmail.com (D.I.K.); Paul.smeets@wur.nl (P.A.M.S.); frans.kok@wur.nl (F.J.K.); rosalie.dhonukshe-rutten@wur.nl (R.A.M.D.-R.); lisette.degroot@wur.nl (L.C.P.G.M.d.G.); 2 Department of Epidemiology and Biostatistics and the EMGO Institute for Health and Care Research, VU University Medical Center, P.O. Box 7057, 1007 MB Amsterdam, The Netherlands; k.swart@vumc.nl; 3 Division of Internal Medicine, Erasmus University Medical Centre, P.O. Box 2040, 3000 CA Rotterdam, The Netherlands; anke_enneman@hotmail.com (A.W.E.); s.c.vandijk@erasmusmc.nl (S.C.v.D.); n.vandervelde@erasmusmc.nl (N.v.d.V.); 4 Department of Internal Medicine, Section Geriatric Medicine, Academic Medical Centre, Postbus 22660, 1100 DD Amsterdam, The Netherlands; 5 Department of Medical Psychology, Radboud University Medical Centre, Postbus 9101, 6500 HB Nijmegen, The Netherlands; Roy.Kessels@radboudumc.nl; 6 Radboud Alzheimer Centre, Radboud University Medical Centre, Postbus 9101, 6500 HB Nijmegen, The Netherlands; 7 Donders Institute for Brain, Cognition and Behavior, Radboud University, P.O. Box 9101, 6500 HB Nijmegen, The Netherlands; 8 Image Sciences Institute, University Medical Center Utrecht, P.O. Box 85500, 3508 GA Utrecht, The Netherlands

**Keywords:** homocysteine, vitamin B_12_, folate, holotranscobalamin, methylmalonic acid, brain volume, grey matter, white matter

## Abstract

Aim: We investigated cross-sectional associations between circulating homocysteine, folate, biomarkers of vitamin B_12_ status and brain volumes. We furthermore compared brain volumes of participants who received daily folic acid and vitamin B_12_ supplementation with participants who did not. Methods: Participants of the B-PROOF study (*n* = 2919) were assigned to 400 µg folic acid and 500 µg vitamin B_12_, or a placebo. After two years of intervention, T_1_-weighted magnetic resonance imaging (MRI) scans were made in a random subsample (*n* = 218) to obtain grey and white matter volume, and total brain volume (TBV). Plasma homocysteine, serum folate, vitamin B_12_, holotranscobalamin, and methylmalonic acid concentrations were measured. Results: Multiple linear regression analyses showed inverse associations between plasma homocysteine with TBV (β = −0.91, 95% CI −1.85–0.03; *p* = 0.06) and between serum folate and TBV (β = −0.20, 95% CI −0.38, −0.02; *p* = 0.03). No significant associations were observed for serum vitamin B_12_ and holotranscobalamin. Fully adjusted ANCOVA models showed that the group that received B-vitamins had a lower TBV (adjusted mean 1064, 95% CI 1058–1069 mL) than the non-supplemented group (1072, 95% CI 1067–1078 mL, *p* = 0.03). Conclusions: Results were contradictory, with higher Hcy levels associated with lower TBV, but also with higher folate levels associated with lower TBV. In addition, the lack of a baseline measurement withholds us from giving recommendations on whether folic acid and vitamin B_12_ supplementation will be beneficial above and beyond normal dietary intake for brain health.

## 1. Introduction

Elevated homocysteine (Hcy) levels have been associated with faster cognitive decline, cognitive impairment and dementia [1], by neurotoxicity or via other, probably vascular, pathways [2]. Remethylation of Hcy into methionine is dependent of vitamin B_12_ and folate. Low intake or low status of these vitamins can result in elevated Hcy levels, and as such, negatively affect cognitive health. A direct negative effect of low vitamin B_12_ status is also possible, as is for instance observed in people with a vitamin B_12_ deficiency, resulting in neurological problems. A low vitamin B_12_ status is characterized by low levels of serum B_12_ and holotranscobalamin (holoTC), and high levels of methylmalonic acid (MMA) and Hcy [3].

Observational studies have shown associations between vitamin B_12_ and folate with cognitive performance. Most of the intervention studies, however, did not show an effect of supplementation with vitamin B_12_ and folic acid on cognitive performance, despite a lowering effect on Hcy concentrations [4]. A possible explanation of the lack of findings after supplementation is that the classical neuropsychological paper-and-pencil tests are not sensitive enough to detect subtle changes in cognitive performance induced by supplementation. Another, relatively new, method to investigate the role of vitamin B_12_, folate and Hcy in brain health, is by studying brain volumes. Cognitive decline has been associated with brain atrophy as measured by magnetic resonance imaging (MRI) [5]. Furthermore, associations or effects of nutrients observed in structural MRI scans may be expected to have long-term consequences on central nervous system functions [6]. Until now, only a few studies have investigated the associations of Hcy, vitamin B_12_ and folic acid with brain volumes. Cross-sectional studies have shown positive associations of Hcy levels with ventricle-brain ratios [2] and white-matter lesions as an index of the integrity of white matter [7]. Additionally, inverse associations were observed between vitamin B_12_ status and white-matter lesions [7,8]. Positive associations were observed between vitamin B_12_ status and total brain volume, cross-sectionally [2,9] and prospectively [10]. Higher folate levels have been associated with less white matter lesions, but not with more hippocampal or amygdalar volume [11].

We investigated the associations of levels of plasma Hcy, serum folate and three markers related to vitamin B_12_ status (serum B_12_, MMA, and holoTC) with volumes of grey and white matter, and total brain volume as a derivative, measured by MRI in the B-PROOF study. We also studied the difference between participants who received a daily supplement with folic acid and vitamin B_12_ for two years and those who did not receive this supplement.

## 2. Materials and Methods

### 2.1. Study Design and Participants

This study was part of the B-PROOF (B-vitamins for the PRevention Of Osteoporotic Fractures) study that was conducted between October 2008 and April 2013 in three research centers in The Netherlands; VU medical center, Erasmus Medical Centre, and Wageningen University. Primary objective of this randomized, double-blind, placebo-controlled intervention study was to assess the efficacy of lowering homocysteine levels, by two years oral supplementation with 400 µg folic acid and 500 µg vitamin B_12_, in the prevention of osteoporotic fractures. Both the placebo tablet and the B-vitamin tablet contained 15 µg (600 IU) vitamin D. Participants were people aged ≥65 years with mildly elevated plasma Hcy levels (12–50 µmol/L). Participant selection has been extensively described elsewhere [12,13]. MRI scans were made in a random subsample of the population of Wageningen University.

**Figure 1:**
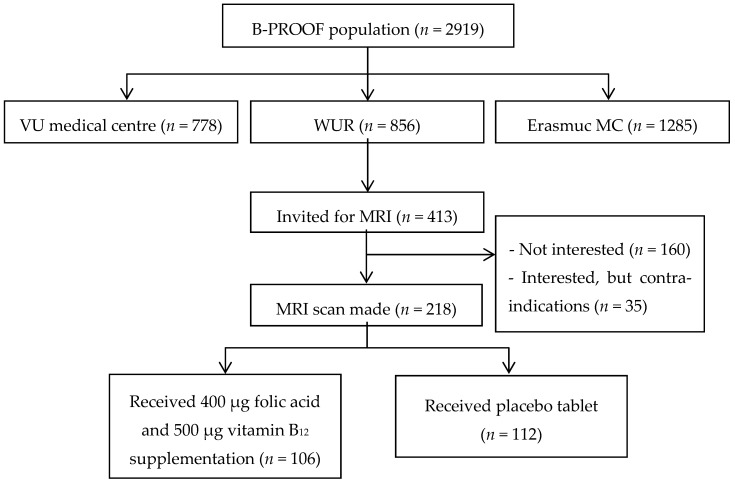
Flowchart of participants that were enrolled in the magnetic resonance imaging (MRI) study, a sub-study of the B-PROOF trial.

Participants who came for their two-year follow-up measurement between July 2012 and April 2013 and who had not dropped out during the intervention period were invited to participate in the MRI study (see Figure 1 for the participant flow). MRI scans were made within one month after completion of the intervention period, and before unblinding the treatment allocation. Participants were carefully screened on contra-indications for MRI. This study was conducted according to the guidelines laid down in the Declaration of Helsinki and all procedures involving human participants were approved by the Wageningen University Medical Ethical Committee. All participants gave their written informed consent. This trial is registered at clinicaltrials.gov as NCT00696514 and at Netherlands Trial Register as NTR1333.

### 2.2. Descriptive Measures

Global cognitive performance was measured with the Mini-Mental State Examination (MMSE) [14]. Depressive symptoms were assessed with the 15-item Geriatric Depression Scale (GDS-15) [15], in which a score ≥5 is indicated as being likely to be depressed. Standing height was measured with a stadiometer to the nearest 0.1 cm and weight was measured to the nearest 0.5 kg with a calibrated scale (Seca, Deventer, The Netherlands). Body mass index (BMI) was calculated by dividing the weight by the squared height (kg/m^2^). Information about highest educational level, smoking habits, alcohol intake (Garrett index [16]), marital status, and living situation was obtained by structured questionnaires. Physical activity was measured with the LASA Physical Activity Questionnaire (LAPAQ) [17]. Self-reported frequency and duration of activities during the past two weeks were checked by a research assistant and were used to calculate physical activity in kcal/day.

### 2.3. MRI Scans

Cranial volumetric MRI scans were made after two years of intervention at the Hospital Gelderse Vallei (Ede, The Netherlands) on a 3-Tesla Siemens Magnetom Verio (Siemens, Erlangen, Germany), with a 32-channel head coil. Here, we analyzed the T_1_-weighted scan (MPRAGE, repetition time = 2300 ms, echo time = 3.0 ms, inversion time = 900 ms, 9° flip angle, field of view = 256 × 256 mm, 192 sagittal slices, voxel size = 1 × 1 × 1 mm, acceleration factor (GRAPPA) = 2).

The voxel-based morphometry (VBM8) toolbox within the SPM8 software (Wellcome Department of Imaging Neuroscience, London, UK) and FSL-VBM v6.0 (FMRIB Software Library, Oxford, UK) [18] were used for segmentation. T_1_-weighted images were first reoriented to match the standard template images in FSL-VBM. VBM8 spatially normalizes participants’ brain images to a standard space, and then automated segments into grey matter, white matter, and cerebrospinal fluid, using a unified tissue segmentation approach [19]. These three measures were summed to calculate intracranial volume. Grey and white matter volumes were summed to calculate total brain volume.

### 2.4. Blood Measurements

Blood samples were obtained in the morning, when participants were fasted or had consumed a restricted breakfast. For the current study, follow-up levels of serum folate and vitamin B_12_ biomarkers were used. Plasma Hcy was measured using the HPLC method (intra assay CV = 3.1%, inter assay CV = 5.9%). Serum vitamin B_12_ and serum folate were analyzed by using electrochemiluminescence immunoassay (Elecsys, 2010, Roche GmbH, Mannheim, Germany) (CV vitamin B_12_ 5.1% at 125 pmol/L and 2.9% at 753 pmol/L; CV folate: 5.9% at 5.7 nmol/L and 2.8% at 23.4 nmol/L). Serum holoTC was determined by the AxSYM analyser (Abbott Diagnostics, Hoofddorp, The Netherlands) (intra assay CV < 8%) and serum MMA was measured by LC-MS/MS (intra assay CV = 8.1% at 0.18 μmol/L, inter assay CV = 1.6% at 0.24 μmol/L). DNA was isolated from buffy coats to determine the genotype for methylenetetrahydrofolate reductase (MTHFR 677TT) using the Illumina Omni-express array and to determine Apolipoprotein E (ApoE) genotype by using Taqman. All analyses were done in the biochemical laboratory of Erasmus MC, except Hcy, which was measured at Wageningen University.

### 2.5. Statistics

Population characteristics are reported as *n* (%), means ± SD, or as median (interquartile range; IQR) for non-normally distributed data. Comparisons between treatment groups were made using Chi-square test, independent-samples *t*-test or Mann–Whitney non-parametric test.

Linear regression analyses were used to examine the associations between serum folate, different markers of vitamin B_12_ status (serum B_12_, holoTC, MMA), and plasma Hcy, with volumes of grey and white matter and total brain volume. The crude model was adjusted for intracranial volume, age and sex, and model 1 included the covariates of the first model plus BMI, smoking, alcohol, education, and physical activity.

Differences between treatment groups were analyzed using analyses of covariance (ANCOVA), in which the brain volumes acted as dependent factor, treatment as fixed between-subject factor, and intracranial volume, age and sex as covariates (crude model). Model 1 included the covariates from first model plus BMI, smoking, alcohol, education, and physical activity.

Results are presented as β-coefficients with 95% confidence interval (95% CI) for the regression analyses, and as adjusted means with 95% CI for the ANCOVA analyses. Alpha was set at 0.05 and two-tailed analyses were performed. All statistical analyses were performed using SPSS Statistics v24 (SPSS Inc., Chicago, IL, USA).

## 3. Results

### 3.1. Participants

The general characteristics of the total subgroup at time of the MRI measurement are presented in Table 1. The group that received two years of B-vitamin supplementation (*n* = 106) and the group that did not receive B-vitamins (*n* = 112) included both 57% men. Participants in the B-vitamin group were significantly younger and had a slightly higher MMSE score (age: 72.7 ± 5.3 year; median MMSE 29, IQR 27–30, range 19–30) than the non-supplemented group (age: 74.5 ± 6.3 year, *p* = 0.02; median MMSE 28, IQR 27–29, range 24–30, *p* = 0.09). Concentrations of folate, vitamin B_12_ and holoTC were higher in the group that received B-vitamin supplementation than in the group that did not, whereas MMA and Hcy were lower (all *p*-values < 0.001), as a result of the two-year supplementation.

### 3.2. B-Vitamin Status and Brain MRI Volumes

Fully adjusted linear regression models of biomarkers and brain volumes in the total population (Table 2) showed an inverse association for serum folate with total brain volume (β = −0.20, 95% CI −0.34–−0.02; *p* = 0.03), and a borderline significant association with white matter volume (β = −0.17, 95% CI −0.34–0.01). Furthermore, plasma Hcy was borderline significantly inversely associated with total brain volume (β = −0.91, 95% CI −1.85–0.03). No significant associations were observed for serum B_12_, holoTC, or MMA with any of the brain volumes in the fully adjusted models.

Associations differed between the group that received B-vitamin supplementation and the group that did not (Table 2), more specifically, interaction terms with treatment were significant or tended towards significance for MMA and grey matter (*p* = 0.04), MMA and total brain volume (*p* = 0.10), and Hcy and total brain volume (*p* = 0.10). In both groups, serum MMA was inversely associated with total brain volume, but only in the group that received B-vitamin supplementation the association remained significant after adjustment for covariates (B-vitamin group: β = −127.1, 95% CI −232.2–−21.9; *p* = 0.02; group without B-vitamins: β = −27.1, 95% CI −57.7, 3.5). Only in the B-vitamin group, MMA was also associated with grey matter volume (β = −136.2, 95% CI −241.6–−30.8; *p* = 0.01). Inverse associations were observed for Hcy and total brain volume in both groups, with stronger associations in the supplemented group (β = −4.9, 95% CI −6.73–−1.44; *p* < 0.01) than in the non-supplemented group (β = −1.88, 95% CI−3.21–−0.55; *p* = 0.01). In the B-vitamin group, a trend was observed for Hcy and grey matter volume (β = −2.53, 95% CI −5.24–0.19), but not for white matter volume. In the non-supplemented group, a trend was observed for Hcy and white matter volume (β = −1.30, 95% CI −2.50–0.10), but not for grey matter volume.

### 3.3. Differences between Supplementation Groups

Table 3 presents the differences in brain volumes between the group that received B-vitamin supplementation and the group that did not. Unadjusted analyses did not show a difference between groups (data not shown). The fully adjusted model, however, revealed a lower total brain volume in the B-vitamin group (1063.6, 95% CI 1058.2–1069.0 mL) compared to the non-supplemented group (1072.3, 95% CI 1067.2–1077.5 mL; *p* = 0.03). This was also reflected by a non-significant (*p* = 0.07) lower volume of white matter in the B-vitamin group (490.2, 95% CI 485.2–495.3 mL) compared to the non-supplemented group (496.7, 95% CI 491.7–501.6 mL). Grey matter volume did not differ between groups.

## 4. Discussion

Our cross-sectional analyses showed that higher levels of Hcy and MMA were associated with lower total brain volumes, with stronger associations in the B-vitamin group than in the non-supplemented group. No associations were observed for serum vitamin B_12_ or holoTC with brain volumetric measures. Higher levels of folate, however, were also associated with lower brain volume. Furthermore, directly comparing the supplementation groups with respect to brain volumes does not point towards a beneficial effect of B-vitamin treatment; after adjustment for important covariates the group that received the B-vitamin supplementation had a lower brain volume compared to the placebo group.

Our data add to two other findings on B-vitamins and brain health within the B-PROOF study. Cross-sectionally, we observed baseline associations between MMA, Hcy and folate, indicating a better vitamin B_12_ and folate status, with episodic memory and information processing speed [20]. Two-year supplementation with folic acid and vitamin B_12_, however, did not show beneficial effects on specific cognitive function domains [21]. Most other randomized controlled trials (RCTs) investigating the effects of B-vitamin supplementation with at least one-year follow-up also failed to show a beneficial effect on cognitive performance [22,23]. Potentially, study populations were not sensitive enough to induce an effect, study durations were too short to significantly slow down the development of cognitive decline, or the effects were too subtle to be detected by the neuropsychological testing. Neuropsychological tests may be susceptible to practice effects or short-term fluctuations in test performance [24], while structural MRI may be less susceptible to these fluctuations. Brain volume can act as a predictor for brain health [24,25,26]; rate of brain volume loss is correlated with performance on cognitive tasks and it may predict the conversion of mild cognitive impairment (MCI) into dementia [5,26,27]. MRI measures can thus be of added value to neuropsychological tests. For instance, within the B-PROOF study, significant positive associations were observed for vitamin D status, glucose levels and grey matter volume [28]. This added to earlier seen significant associations of vitamin D status and cognitive functioning [29].

We observed a negative association between folate and brain volume in the total population. After stratification, the cross-sectional association was not present in the group that did not receive B-vitamin supplementation, whereas a trend was still seen in the supplemented group. The non-supplemented group may reflect a sample with blood levels closer to normal instead of being intervened by folic acid supplementation, and thus it might be that these associations come closer to normal values. Folic acid, the synthetic form used in supplements, has a higher bioavailability than folate naturally present in food. The conversion of folic acid to metabolized folate is low because of low activity of the enzyme dihydrofolate reductase, and therefore high intake of synthetic folic acid may cause an accumulation of unmetabolized folic acid. Studies have suggested that high folate levels, in combination with and without vitamin B_12_ deficiency can be detrimental for cognitive health [30,31,32]. Especially older adults who took folic acid supplements containing >400 µg folic acid supplements, had a higher risk for cognitive decline [32]. Hence, it is possible that folate levels follow an inverted U-shape regarding optimal cognitive performance and brain health; cross-sectional studies and intervention studies, however, are inconclusive in their findings [11,33,34]. Another point to address is that serum folate, as used in the present study, reflects more recent intake, while red blood cell folate concentrations seem to be a more robust biomarker of folate status on the longer term [35]. It would be worth examining the association between this biomarker and brain volumes. Unfortunately, we do not have data on unmetabolized folic acid or on red blood cell folate concentrations.

Our cross-sectional findings regarding Hcy and vitamin B_12_ status and brain volumes are similar to other studies. Studies that investigated Hcy concentrations and brain atrophy showed clear associations, with higher levels associated with more total atrophy [2,36,37] and lower hippocampal volume [38]. In line with our findings, associations of MMA and Hcy with total brain volume measured 4.6 years later have been shown, but not with serum vitamin B_12_ [9]. A prospective study (*n* = 107, mean age 73 year), however, showed that lower serum concentrations of vitamin B_12_ and holoTC, but not folate, Hcy or MMA, were associated with smaller brain volumes after five years of follow-up [10]. The only RCT currently published was performed in 168 patients with MCI, showing that B-vitamin (B_12_, B_6_ and folic acid) supplementation slowed down total brain atrophy and grey matter atrophy, but only in those with the highest Hcy levels [39]. We observed a borderline significant association between Hcy and grey matter in our healthier study population, but only in the group that received B-vitamin supplementation. Interestingly, the observed associations were thus stronger in those with lower Hcy levels as a result of the B-vitamin treatment. It might be that the stronger associations between vitamin B_12_ status, as reflected by Hcy and MMA, and brain volumes in the B-vitamin group are the results of the intervention, but because baseline MRI data are lacking, no conclusions can be drawn about the causality of the relation between the intervention and the MRI measures.

In contrast to our hypothesis, we observed that participants who received B-vitamin supplementation had lower brain volumes than the non-supplemented group. Participants who received B-vitamins were significantly younger than participants who did not receive the supplements. Age is a major predictor for brain atrophy [40] and it is possible that our findings may be the result of this two-year age difference between the two groups. This hypothesis is supported by the fact that the unadjusted model did not show differences on brain volumes between treatment groups, whereas a difference was expected due to the age difference (data not shown). When adjustments for age were made, this resulted in a significant difference in brain volume between the two groups.

The observed cross-sectional associations of higher Hcy concentrations and lower brain volume may be explained by several mechanisms. Hcy may be neurotoxic, which can induce brain atrophy and hamper neurogenesis [2,36]. Furthermore, elevated Hcy levels may lead to an increase of phosphorylated tau, which is present in neurofibrillary tangles and is associated with atrophy in specific brain regions [41,42]. Vascular pathways have also been suggested, based on negative associations between Hcy and vascular health and white matter hyperintensities [8,43]. An elevated Hcy level as indicator of low vitamin B_12_ status may have influence on myelin and consequently on the integrity of white matter, as vitamin B_12_ deficient patients show areas of demyelination on brain MRI [44,45].

To put our findings into perspective, some methodological issues need to be discussed. Limitations include the lack of baseline data on brain volumes, which makes it impossible to draw conclusions on the effects of two years B-vitamin supplementation on grey and white matter volume. Furthermore, one of the inclusion criteria of the B-PROOF study was an elevated Hcy level, which makes the generalizability to the total elderly population difficult. Last, we performed multiple statistical tests, which increased the risk on chance findings. Strengths of our study are the use of a 3T scanner to make high-precision images, our large study population for conducting MRI scans, the possibility to adjust for multiple confounders, and the available data of folate, Hcy and three markers of vitamin B_12_ status. Furthermore, previous data analyses in the total B-PROOF population showed that supplement use was very low at baseline. Vitamin B_12_ derived from diet was the main source that contributed to baseline serum vitamin B_12_ levels [46]. At follow-up, i.e., at the time MRI scans were performed, we also did not observe differences in self-initiated supplement use between treatment groups in the subpopulation of the current paper. Questionnaires to assess supplement usage, however, may not be reliable for a long-term micronutrient intakes in older adults [47].

## 5. Conclusions

To conclude, the results suggest that lower levels of Hcy and the vitamin B_12_-metabolite MMA may be important in order to attenuate brain atrophy in healthy elderly people. However, the negative associations of folate and the lack of a baseline measurement withhold us from giving recommendations on whether folic acid and vitamin B_12_ supplementation will be beneficial above and beyond normal dietary intake for brain health. Furthermore, the age difference between the groups may have distorted the results. More research, especially well-designed randomized controlled trials with a sufficient follow-up time, is required to unravel the role of folate levels, in combination with and without low vitamin B_12_ levels, in brain health.

## Figures and Tables

**Table 1:** Population characteristics at time of MRI measurements in participants who received two years of B-vitamin supplementation and participants who did not receive this supplementation.

Variable	Total Population (*n* = 218)	With B-Vitamin Supplementation (*n* = 106)	Without B-Vitamin Supplementation (*n* = 112)	*p*-Value
Age	73.6 ± 5.9	72.7 ± 5.3	74.5 ± 6.3	0.02
Sex, men, *n* (%)	124 (57%)	60 (57%)	64 (57%)	0.94
Body mass index, kg/m^2^	27.7 ± 4.2	27.3 ± 4.4	28.1 ± 4.1	0.21
Blood pressure, systolic, mmHg	147 ± 18	146 ± 19	148 ± 17	0.42
Blood pressure, diastolic, mmHg	78 ± 10	78 ± 10	78 ± 10	0.76
Education, *n* (%)				0.12
Low	90 (41%)	49 (46%)	41 (37%)	
Medium	52 (24%)	19 (18%)	33 (30%)	
High	76 (35%)	38 (36%)	39 (34%)	
Smoking, *n* (%)				0.67
Never	64 (29%)	27 (26%)	37 (33%)	
Former	139 (64%)	70 (66%)	69 (62%)	
Current	15 (7%)	9 (9%)	6 (5%)	
Alcohol, *n* (%)				0.72
Light	152 (70%)	73 (69%)	79 (71%)	
Moderate	60 (28%)	31 (29%)	29 (26%)	
Excessive	6 (3%)	2 (2%)	4 (3%)	
Self-initiated supplement use, vitamin B_12_, *n* (%)	27 (8%)	11 (7%)	16 (10%)	0.43
Self-initiated supplement use, folic acid, *n* (%)	30 (12%)	12 (9%)	18 (15%)	0.37
MMSE, max 30 points	28 [27–29]	29 [27–30]	28 [27–29]	0.09
Low MMSE < 25, *n* (%)	6 (3%)	4 (4%)	2 (2%)	0.37
GDS, max 15 points	1 (0–2)	1 (0–2)	1 (0–2)	0.89
Physical activity (kcal/day)	664 ± 416	642 ± 288	683 ± 440	0.47
ApoE-ε4, carrier, *n* (%)	60 (28%)	32 (30%)	28 (25%)	0.45
MTHFR, 677TT, *n* (%)	28 (13%)	17 (15%)	11 (10%)	0.40
Grey matter (mL)	574 ± 56	577 ± 57	572 ± 55	0.51
Grey matter/ICV	0.42 ± 0.02	0.42 ± 0.02	0.42 ± 0.02	0.98
White matter (mL)	493 ± 61	495 ± 63	492 ± 60	0.73
White matter/ICV	0.36 ± 0.02	0.36 ± 0.02	0.36 ± 0.02	0.74
Cerebrospinal fluid (mL)	304 ± 52	306 ± 51	302 ± 53	0.62
Cerebrospinal fluid/ICV	0.22 ± 0.03	0.22 ± 0.03	0.22 ± 0.03	0.81
Serum folate (nmol/L)	36.7 [23.4–54.2]	53.1 [42.5–68.3]	24.1 [19.5–31.8]	<0.001
Serum vitamine B_12_ (pmol/L)	404 [262–558]	558 [459–715]	274 [222–373]	<0.001
Vitamin B_12_ < 258 pmol/L, *n* (%)	49 (23%)	1 (1%)	48 (43%)	<0.001
Serum holotranscobalamin (pmol/L)	81 [56–111]	111 [89–147] ^a^	63 [44–80]	<0.001
Holotranscobalamin < 30 pmol/L, *n* (%)	12 (6%)	1 (1%)	11 (10%)	0.01
Serum methylmalonic acid (µmol/L)	0.20 [0.16–0.25]	0.17 [0.15–0.21]	0.24 [0.19–0.30]	<0.001
Methylmalonic acid > 0.30 µmol/L, *n* (%)	35 (16%)	5 (5%)	30 (27%)	<0.001
Plasma homocysteine (µmol/L)	11.4 [8.9–14.4]	9.1 [7.8–10.6] ^b^	13.9 [12.0–16.3] ^c^	<0.001

Values are reported as mean ± SD, median [interquartile range], or as *n* (%); MMSE, Mini-Mental State Examination; GDS, Geriatric Depression Scale; ApoE-ε4, Apolipoprotein E; MTHFR, methylenetetrahydrofolate reductase; ICV, intracranial volume; ^a^ *n* = 96; ^b^ *n* = 103; ^c^ *n* = 109.

**Table 2:** Associations between follow-up blood values and brain MRI measures in the total population (*n* = 218) and stratified for the group with (*n* = 106) and without a history of B-vitamin supplementation (*n* = 112) (β-coefficients (95% CI confidence interval).

Variable	Total Population (*n* = 218)		Without B-Vitamin Supplementation (*n* = 112)		With B-Vitamin Supplementation (*n* = 106)	
	Crude	Model 1	Crude	Model 1 ^f^	Crude	Model 1 ^i^
**Serum folate, nmol/L**						
Grey matter	−0.05 (−0.22, 0.13)	−0.03 (−0.21, 0.15) ^a^	0.29 (−0.18, 0.75)	0.19 (−0.28, 0.65)	−0.07 (−0.38, 0.24)	−0.03 (−0.36, 0.31)
White matter	−0.14 (−0.31, 0.02)	−0.17 (−0.34, 0.01) ^a^	−0.08 (−0.50, 0.34)	−0.06 (−0.49, 0.37)	−0.14 (−0.43, 0.16)	−0.24 (−0.56, 0.09)
Total brain volume	−0.19 (−0.37, −0.01)	−0.20 (−0.38, −0.02) ^a,^*	0.20 (−0.27, 0.68)	0.11 (−0.46, 0.68)	−0.20 (−0.51, 0.11)	−0.24 (−0.57, 0.09)
**Serum vitamin B_12_, pmol/L**						
Grey matter	−0.01 (−0.02, 0.01)	−0.01 (−0.02, 0.01)	0.02 (−0.03, 0.06)	0.01 (−0.04, 0.06)	−0.01 (−0.04, 0.02)	−0.01 (−0.04, 0.02)
White matter	0.00 (0.01, 0.02)	−0.00 (−0.02 0.02)	0.02 (−0.02, 0.07)	0.02 (−0.02, 0.07)	0.02 (−0.01, 0.04)	0.02 (−0.01, 0.04)
Total brain volume	−0.01 (−0.02, 0.01)	−0.01 (−0.03, 0.01)	0.04 (−0.01, 0.09)	0.03 (−0.02, 0.08)	0.01 (−0.02, 0.04)	0.00 (−0.02, 0.03)
**Serum holotranscobalamin, pmol/L**						
Grey matter	−0.00 (−0.09, 0.09) ^b^	0.01 (−0.08, 0.09) ^c^	0.00 (−0.21, 0.21)	−0.04 (−0.25, 0.18)	0.05 (−0.09, 0.18) ^j^	0.06 (−0.08, 0.20) ^k^
White matter	0.02 (−0.06, 0.10) ^b^	0.01 (−0.07, 0.09) ^c^	0.15 (−0.04, 0.34)	0.16 (−0.04, 0.35)	0.06 (−0.07, 0.19) ^j^	0.05 (−0.09, 0.18) ^k^
Total brain volume	0.02 (−0.07, 0.11) ^b^	0.02 (−0.07, 0.10) ^c^	0.16 (−0.06, 0.37)	0.12 (−0.10, 0.34)	0.10 (−0.03, 0.24) ^j^	0.11 (−0.03, 0..24) ^k^
**Serum methylmalonic acid, µmol/L**						
Grey matter	−13.2 (−39.3, 12.9)	−14.5 (−40.6, 11.5) ^a^	−8.9 (−38.7, 20.8)	−10.5 (−40.5, 19.5)	−136.6 (−237.2, −36.0) *	−136.2 (−241.6, −30.8) *
White matter	−7.7 (−32.2, 16.8)	−7.2 (−32.3, 17.9) ^a^	−19.9 (−46.8, 6.0)	−16.7 (−44.3, 10.9)	29.9 (−70.1, 130.2)	9.4 (−98.0, 116.9)
Total brain volume	−20.9 (−47.4, 5.6)	−21.6 (−48.1, 4.8) ^a^	−28.9 (−58.7, 0.90)	−27.1 (−57.7, 3.5)	−106.7 (−208.8, −4.5) *	−127.1 (−232.2, −21.9) *
**Plasma homocysteine, µmol/L**						
Grey matter	−0.33 (−1.26, 0.60) ^d^	−0.53 (−1.47, 0.41) ^e^	−0.67 (−1.98, 0.64) ^g^	−0.78 (−2.09, 0.53) ^h^	−1.88 (−4.47, 0.70) ^i^	−2.53 (−5.24, 0.19) ^L^
White matter	−0.36 (−1.22, 0.51) ^d^	−0.43 (−1.31, 0.50) ^e^	−1.08 (−2.23, 0.07) ^g^	−1.30 (−2.50, −0.10) ^h,^*	−2.10 (−4.58, 0.39) ^i^	−1.19 (−3.87, 1.49) ^L^
Total brain volume	−0.69(−1.62, 0.25) ^d^	−0.91 (−1.85, 0.03) ^e^	−1.75 (−3.02, −0.48) ^g,^*	−1.88 (−3.21, −0.55) ^h,^**	−3.98 (−6.47, −1.49) ^i,^**	−4.09 (−6.73, −1.44) ^L,^**

Crude: Adjusted for intracranial volume, age, sex; Model 1: Adjusted for model 1 and BMI, alcohol, smoking, education, physical activity; ^a^ *n* = 214; ^b^ *n* = 208; ^c^ *n* = 205; ^d^ *n* = 212; ^e^ *n* = 208; ^f^ *n* = 111; ^g^ *n* = 109; ^h^ *n* = 108; ^i^ *n* = 103; ^j^ *n* = 96; ^k^ *n* = 94; ^L^ *n* = 100. * *p* < 0.05; ** *p* < 0.01.

**Table 3:** Differences in brain volumes (mL) between participants who received two-year B-vitamin supplementation (*n* = 106) and participants who did not receive this supplementation (*n* = 112) (adjusted means (95% confidence interval).

	Crude Model			Model 1 ^a^		
	Without B-Vitamin Supplementation	With B-Vitamin Supplementation	*p*-Value	Without B-Vitamin Supplementation	With B-Vitamin Supplementation	*p*-Value
Grey matter	575.7 (570.6, 580.8)	572.5 (567.3, 577.8)	0.40	575.6 (570.4, 580.8)	573.4 (568.0, 578.7)	0.57
White matter	495.6 (490.8, 500,4)	490.3 (485.4, 495.2)	0.13	496.7 (491.7, 501.6)	490.2 (485.2, 495.3)	0.07
Total brain volume	1071.3 (1066.1, 1076.5)	1062.8 (1057.5, 1068.1)	0.03	1072.4 (1067.2, 1077.5)	1063.6 (1058.2, 1069.0)	0.03

Differences between groups were tested with ANCOVA, adjusted for intracranial volume, age, sex (crude model), BMI, alcohol, smoking, education, and physical activity (model 1); ^a^ *n* = 111 for the group without B-vitamin supplementation, *n* = 103 for the group with B-vitamin supplementation.
